# Microstructural and Mechanical Properties of Alkali Activated Colombian Raw Materials

**DOI:** 10.3390/ma9030158

**Published:** 2016-03-05

**Authors:** Maria Criado, Willian Aperador, Isabel Sobrados

**Affiliations:** 1 Instituto de Ciencia de Materiales de Madrid (ICMM), CSIC, Sor Juana Inés de la Cruz 3, Cantoblanco, Madrid 28049, Spain; mcriado@icmm.csic.es (M.C.); isobrado@icmm.csic.es (I.S.); 2 School of Engineering, Universidad Militar Nueva Granada, Carrera 11 #101-80, Bogotá 49300, Columbia

**Keywords:** alkaline activation, slag and fly ash, mineralogical and microstructural characterization, mechanical properties

## Abstract

Microstructural and mechanical properties of alkali activated binders based on blends of Colombian granulated blast furnace slag (GBFS) and fly ash (FA) were investigated. The synthesis of alkali activated binders was conducted at 85 °C for 24 h with different slag/fly ash ratios (100:0, 80:20, 60:40, 40:60, 20:80, and 0:100). Mineralogical and microstructural characterization was carried out by means of X-ray diffraction (XRD), Fourier transform infrared spectroscopy (FTIR), Scanning electron microscopy with energy dispersive X-ray spectroscopy (SEM/EDX) and Nuclear magnetic resonance (NMR). Mechanical properties were evaluated through the compressive strength, modulus of elasticity and Poisson’s ratio. The results show that two different reaction products were detected in the slag/fly ash mixtures, a calcium silicate hydrate with Al in its structure (C-A-S-H gel) and a sodium aluminosilicate hydrate (N-A-S-H gel) with higher number of polymerized species and low content in Ca. It was found that with the increase of the amount of added slag, the amount of C-A-S-H gel increased and the amount of N-A-S-H gel decreased. The matrix was more dense and compact with almost absence of pores. The predominance of slag affected positively the compressive strength, Young’s modulus and Poisson’s ratio, with 80% slag and 20% fly ash concrete being the best mechanical performance blend.

## 1. Introduction

World ordinary Portland cement (OPC) production, which is conventionally used as the primary material for the manufacturing of concrete, was in recent years about 1.8 billion Metric tons  with an approximate consumption of three billion Metric tons of natural resources as raw materials (limestone and clay) and fossil fuels, and a significant energy demand (3530 kJ/tonne cement). Concrete is the second most consumed substance on Earth after water; its production was about four million Metric tons in 2014 [1]. In addition to cement; eight million Metric tons of aggregates and between 2270 and 2650 billion liters of water were consumed in its manufacturing. It should be noted that the manufacture of one tonne of cement produces around one tonne of CO_2_ as well as SO_x_ and NO_x_ and that the cement industry is considered responsible for 6%–7% of all greenhouse gases emitted worldwide. Fifty percent of CO_2_ comes from the decarbonation of limestone in the clinkering process, and the remainder is attributed to the burning of fossil fuels [2,3]. One effort to combat these problems is the development of alternative binders to OPC aiming to reduce the environmental impact of construction, using a greater proportion of waste pozzolan, and also to improve concrete performance. A possible alternative are the new binders known as alkali activated materials.

Alkali activated binders are made by mixing industrial aluminosilicate waste materials such as class F fly ash (FA) or ground blast furnace slag (GBFS) with an alkaline activating solution. FA is a waste material generated by the coal combustion in thermal power plants and GBFS is a byproduct of iron production in steel plants. The main reaction product of alkali activated fly ash, which is rich in Si and Al, is N-A-S-H gel (Na_2_O-Al_2_O_3_-SiO_2_-H_2_O) with a three-dimensional framework of SiO_4_ and AlO_4_ tetrahedra linked through shared O atoms [4,5,6,7,8], whereas the main reaction product of alkali activated GBFS, which is rich in Ca, is C-S-H gel (CaO-SiO_2_-H_2_O) with a low Ca/Si ratio (0.8–1.1) [9,10,11,12,13]. Recently, the synthesis of alkali activated binders based on FA and GBFS blends has become very attractive, because joint activation of both waste materials can counterbalance the disadvantages that each of the raw materials exhibit when alkali activated separately [14,15,16,17,18,19].

The alkali rate and chemical composition of the reaction products depend on factors such as particle size and chemical composition of raw materials, type and concentration of activator, curing conditions (temperature, time and relative humidity), solution/binder ratio and so on. If we focus on the nature of the starting materials, there are factors that affect their capacity to be alkaline activated. Among them, the most important ones are: percentage of unburned material (acting as inert particles and being responsible for increasing the liquid/solid ratio), particle size distribution, specific surface and content of vitreous particles. The greater the percentage of fine particles (lower than 45 µm), the higher the compressive strengths. In addition, the higher amount of glassy constituent in the raw materials, the faster the activation process and the greater the degree of reaction. On the other hand, the amount of reactive silica is very important because reactive silica is the part of the fly ash reacting with the alumina and the alkalis to give the alkaline aluminosilicate hydrate gel and consequently a high mechanical strength is developed in the cementitious material. The slag must be granular or pelleted with basic nature CaO/SiO_2_ + Al_2_O_3_ > 1, as the lime content of the slag controls the activation process.

All these individual characteristics of the starting materials of alkaline activation process has been discussed [20,21,22,23], being impossible to determine which factor is most important, but considering that all will influence on the mechanical behavior of the final material. Binders based on alkali activated FA-GBFS blends show higher compressive strength comparing to the strength of sole alkali activated FA. The dominant factor in the strength gain of the FA-GBFS binders is the amount of slag in the blend [24,25,26]. The modulus of elasticity of OPC concrete was 15%–29% higher than that observed for alkaline concrete [27]. The modulus of elasticity increased as the compressive strength of geopolymer concrete increased [28]. Diaz-Loya *et al.* [29] observed that the values of Poisson’s ratio of the geopolymer concrete appeared to reside toward the low end of range compared to typical values of OPC concrete.

In this work, mineralogical, microstructural and mechanical characterizations of several alkali activated mixtures of slag and fly ash have been carried out. The difference in the mechanical properties stems from the different microstructures and compositions of the reaction products. The final aim of this study is to determine the optimum percentage of slag and fly ash to obtain the binder with the best mechanical performance.

## 2. Experimental

### 2.1. Materials

The materials used in this work are a fly ash (FA) from the power industrial plant Sochagota, Colombia and a granulated blast-furnace slag (GBFS) from the company “Acerías Paz del Rio”, located in Boyacá, Colombia. The chemical compositions of the materials determined by X-ray fluorescence (XRF) are given in Table 1. Table 2 shows the composition of the mixtures of slag and fly ash prepared in this research. The fly ash and slag have a specific weight of 2230 kg m^−3^ and 2800 kg m^−3^, respectively.

### 2.2. Alkali Activation of the Mixtures

The mixtures were activated with 85% 14M NaOH + 15% waterglass solution with “solution/mixture” ratio of 0.55. The products used to prepare the solutions were laboratory grade reagents: PA-ACS-ISO, 98% sodium hydroxide (NaOH) pellets supplied by Panreac (Castellar del Vallès, Barcelona, España ), and waterglass (Na_2_SiO_3_) with the following composition: 9.1% Na_2_O, 27.5% SiO_2_ and 63.4% H_2_O and SiO_2_/Na_2_O ratio of 3.02. The pastes were cured in an oven at 85 °C for 24 h. After the experiment, the material was removed from the stove and cooled to laboratory temperature. At 90 days of the reaction, the pastes were ground and then mixed with small amounts of acetone to prevent the activation progress.

### 2.3. Techniques

The materials were studied for mineral composition and microstructural characteristics with XRD, FTIR, ^29^Si and ^27^Al Magic Angle Spinning (MAS-NMR) and Scanning electron microscopy with energy dispersive X-ray spectroscopy (SEM/EDX). X-ray diffractograms of powder samples were recorded on a Bruker D8 Advance with a Sol-X detector (Karlsruhe, Germany). X-ray analysis was run in a 2θ range from 5 to 65 in the step-scanning mode, step size 0.02° (2θ) and 0.5 s counting time. The FTIR spectra were measured using a Bruker IFS 66V/S (Karlsruhe, Germany) from 4000 to 250 cm^−1^, with a resolution of 2 cm^−1^. The KBr pellet method was used to prepare the samples. Solid-state ^29^Si and ^27^Al MAS-NMR spectra were recorded using a Bruker Avance-400 pulse spectrometer (Karlsruhe, Germany). Spectra were recorded after irradiation of samples with a π/2 (5-μs) pulse. The resonance frequencies used were 79.5 and 104.3 MHz (9.4 T magnetic field). In order to avoid saturation effects, the recycle delay times used was 10 s. The spinning rate used in MAS-NMR experiments was 10 kHz. All measurements were taken at room temperature with TMS (tetramethylsilane) and Al(H_2_O)_6_^+3^ as external standard. The error in chemical shift values was estimated to be lower than 0.5 ppm. NMR spectra deconvolutions were performed using the Dynamic Modelling fit (DMFIT software) [30]. Chemical shift (position of the line), intensity (integrated area), width (width at half height) and line shape (Lorentzian or Gaussian) of components were deduced. A field emission-scanning electron microscopy (FE-SEM ) using a FEI Nova NanoSEM 230 (Hillsboro, OR, USA) equipped with an EDAX Genesis XM2i analyzer (Hillsboro, OR, USA) was used for the microstructural characterization of the samples.

### 2.4. Compressive Strength, Young's Modulus and Poisson’s Ratio

The study of compressive strength, Young’s modulus and Poisson’s ratio were performed on cylindrical specimens with dimensions of 15 × 30 cm^2^. These specimens were prepared by mixing of aggregates, the fly ash and/or slag and the activating solution. The fine aggregate used was a type of river sand with fineness modulus of 3.04 and absorption of 0.81%. The coarse aggregate employed was a fine gravel of gray tones with nominal maximum size of 10 mm. All mixtures were prepared with an alkaline solution/binder ratio of 0.55. Viscocrete 2100 superplasticizer from the company SIKA (Bogotá, Colombia) was used to ensure the settlement and the workability of the mixtures.

In the manufacturing process, aggregate and cementitious materials were dry blended with the help of a concrete mixer for five minutes, then alkaline activator and liquid plasticizer were added, and the mixing process was continued for other five minutes. Each fresh mixture was poured into molds, tamped down and compacted by traditional methods. The mixtures with different contents of the slag and fly ash were kept in a stove at 85 °C for 24 h and subsequently these specimens were demolded and stored at room temperature until the day of trial.

Compressive strength was determined from specimens into cylinders at ages 28 and 90 days following the procedure of ASTM C39/C39M-15A [31]. The load was applied using a hydraulic press controlled by an automated system; the load application speed was 0.25 MPa/s. For a correct application of the load, metal and neoprene discs were used.

The modulus of elasticity or Young’s modulus and Poisson’s ratio were determined from cylindrical specimens with the ASTM C469/C469M-14 [32]. In tests, a compressometer–extensometer device was used, constituted by three rings articulated pivots to keep the distances between each ring. Two analog gages are coupled to the device and they are responsible for registering changes in the length (longitudinal displacement) and diameter (transverse displacement) of the specimen when subjected to compression by an axial force. Additionally, in each specimen, strain gauges adhered in longitudinal direction for feedback measurements compare the modulus values obtained by the two methods (Compressometer *vs.* strain gauges). To determine the elastic modulus and Poisson's ratio, initially a load of 40% of the maximum compressive strength was applied. To register the full stress–strain curve and observe the ability of maximum deformation and ductility of each specimen in compression the compressometer is retired.

## 3. Results and Discussion

### 3.1. Characterization of the Starting Materials

**Figure 1:**
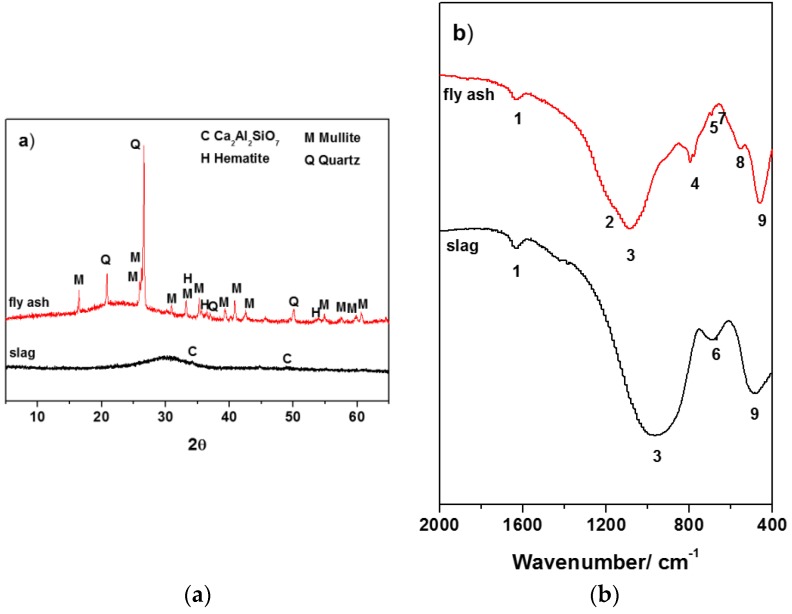
(**a**) XRD patterns; and (**b**) FTIR spectra of the starting materials.

X-ray powder diffraction pattern for the two original materials studied are shown in Figure 1a. Both GBFA and FA consisted mainly of an amorphous phase, indicated by a wide and diffusive reflection in the interval of 25–35° angles 2θ in the case of the slag and in the unreacted fly ash between 2θ = 15–35°. The differences in the location of this hump were associated with the structural differences in the amorphous glasses present in the two raw materials. However, the spectra also contained a series of minority crystalline phases such as Ca_2_Al_2_SiO_7_ (JCPDS 00-034-1236) for the slag and quartz (SiO_2_, JCPDS 00-033-1161), mullite (3Al_2_O_3_-2SiO_2_, JCPDS 00-015-0776) and hematite (Fe_2_O_3_, JCPDS 00-001-1053) for the fly ash.

Figure 1b shows the infrared spectroscopic results for the GBFA and FA. Both spectra exhibited two main very wide and intense bands characteristics of the internal vibrations in TO_4_ tetrahedra (T = Si, Al). One, which peaks at around 966 and 1084 cm^−1^ (peak 3) for the slag and fly ash, respectively, consistent with the differences in the chemical composition and glass structures of these materials, was associated with T-O bond asymmetric stretching vibrations, ν_3_ (Si-O). Low wavenumber of this band was associated with lower degrees of crosslinking of the amorphous phase of the starting materials, induced by increased contents of calcium in their structure. While the other, centered at 488 and 460 cm^−1^ (peak 9) for the slag and fly ash, respectively, corresponded to T-O bond internal deformation vibrations, ν_4_ (O-Si-O) [7,10,33,34]. Low intensity at 1635 cm^−1^ (peak 1) was attributed to deformation vibrations (H-O-H) of water. The Infrared spectroscopy (IR) spectrum of the anhydrous slag also presented a band at 688 cm^−1^ (peak 6), due to asymmetric stretching vibrations generated by the Al-O bonds in the AlO_4_ groups. The presence of quartz in the original ash gave rise in the spectrum to a series of bands located at 1175, 1084, 795–776 (double band), 692, 667, 554 and 460 cm^−1^ (peaks 2, 3, 4, 5, 7, 8 and 9) [7,29]. The bands at around 1180–1130 cm^−1^ (peak 2) and 560–550 cm^−1^ (peak 8, associated with octahedral aluminum) were attributed to mullite [7,35].

**Figure 2:**
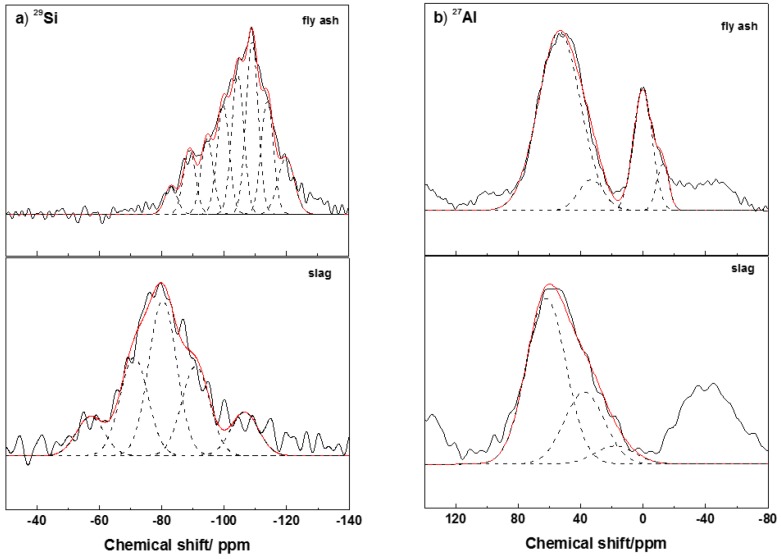
MAS-NMR (**a**) ^29^Si; and (**b**) ^27^Al spectra of the anhydrous slag and fly ash.

^29^Si and ^27^Al MAS-NMR spectra of the anhydrous slag and fly ash appear in Figure 2. The assignment of NMR components was based on reported values obtained in aluminosilicates [15,36,37,38,39,40]. According to published data the peaks appearing between −66 and −73 ppm are assigned to Q^0^ units, between −74 and −78 ppm are assigned to Q^1^ units, between −83 and −88 ppm to Q^2^ units, between −95 and −100 ppm to Q^3^ units and between −103 and −115 ppm to Q^4^ units. The substitution of Si by Al shifts signal 3 or 5 ppm towards more positive values; from this fact the peak appearing at −82 to −84 ppm can be ascribed to Q^2^(1Al).

Figure 2a shows the ^29^Si spectra for the initial GBFS and FA. Their spectra were very wide and poorly defined profiles indicating the heterogeneous distribution of the silicon atoms in these materials. The deconvolution of this spectrum showed the presence of five and eight components for the slag and fly ash respectively. The peaks detected at −57, −71, −80 and −91 were assigned to the glassy component of the slag, while the peaks at −83, −95, −100, −104 and −109 ppm were associated with the initial vitreous material of the ash. Again the differences in the peak position are due to the structural differences in the amorphous glasses of both materials. The peak at −89 ppm corresponded to the crystalline mullite present in the fly ash [41]. Finally, the peaks with chemical shift above −107 ppm were attributed to Q^4^(0Al) environments in quartz (−108/−109 ppm) and other amorphous silica phases [37,38]. The ^27^Al MAS-NMR spectra for both materials (see Figure 2b), had a strong and broad asymmetric signal around +55 ppm formed by components, one centered at +62/+53 ppm associated with the presence of tetrahedrally coordinated aluminum and another smaller centered at +38/+33 ppm due to pentahedral aluminum. A third signal at +18/−2 ppm revealed the presence of octahedrally coordinated aluminum [34,40]. The last component showed higher intensity for the fly ash spectrum, suggesting the presence of a large amount of mullite in this material [42].

### 3.2. Mineralogical and Microstructural Characterization of the Alkali-Activated Binders

**Figure 3:**
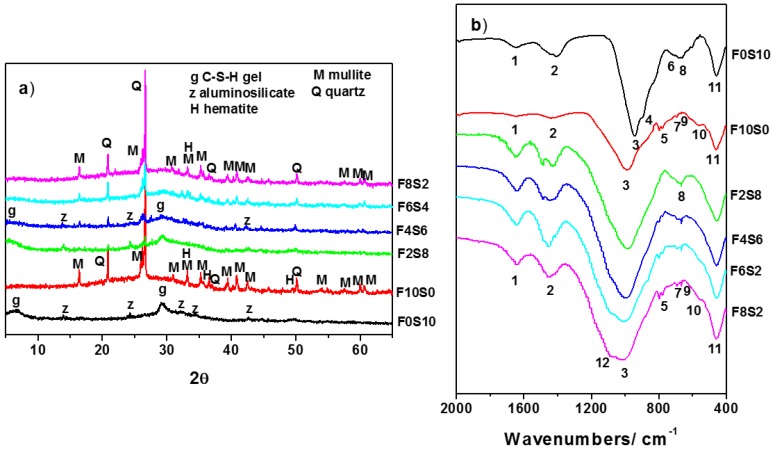
(**a**) XRD patterns; and (**b**) FTIR spectra of the different binders.

Mineralogical analysis carried out through XRD on the pastes at 90 days of reaction showed clear differences in the identified phases of the activated raw materials, see Figure 3a. In the activated slag paste with the alkaline solution (85% 14M NaOH + 15% waterglass), phases identified were sodium aluminosilicate hydroxide hydrate (Na_8_(AlSiO_4_)6(OH)-24H_2_O, JCPDS 00-041-0009) and a C-S-H gel (JCPDS 00-033-0306). In the activated fly ash paste, the halo attributed to the vitreous phase in the initial ash shifts to slightly higher angular values (2θ = 25–40°). This effect is indicating the formation of a sodium aluminosilicate hydrate (N-A-S-H) gel of amorphous nature and three-dimensional network [43]. The crystalline phases (quartz, mullite and hematite) detected in the initial material remained apparently unaltered with the activation. X-ray powder patterns for the alkali activated mixtures showed the formation of different reaction products, which varied depending on the percentage of each raw material, see Figure 3a, but no new phases were detected. In the F2S8 mixture (with 80% of slag and 20% of fly ash), C-S-H gel and aluminosilicate hydrate were clearly detected, whereas diffraction lines corresponding to quartz and mullite from the ash were very small. On increasing the content of the fly ash (from 40% to 80%) implied: (i) a decrease in the amount of C-S-H gel and aluminosilicate hydrate, which were not detected in the F6S4 and F8S2 mixtures; (ii) an increase in the amount of quartz and mullite; and (iii) the width of the halo at 25–40° in 2θ became larger, meaning that the F8S2 mixture presented the highest proportion of N-A-S-H gel.

Figure 3b shows the FTIR spectra of the activated slag, fly ash and mixtures of both materials in different percentages. In the FTIR spectrum of the alkali activated slag, the main absorption bands were as follows: 1647, 1413, 943, 897, 714, 668 and 460 cm^−1^. Conversely, in the spectrum of the activated fly ash, they were: 1643, 1448, 989, 795-776, 695, 667, 554 and 460 cm^−1^. The band T-O at 1084–966 cm^−1^ of the original materials became sharper and shifted towards lower frequencies in the activated slag and in the activated fly ash: 943 and 989 cm^−1^ respectively (see Figure 3b, peak 3). These displacements were indicating that the vitreous component of the slag and fly ash was reacting with the alkali activator and therefore that new products of reaction were being formed (the main ones: calcium silicate hydrate gel and sodium aluminosilicate hydrate gel, respectively) [44,45]. This difference in the wavenumber of asymmetric stretching vibrations for two materials was attributed to the length as well as angle of the Si-O and Al-O bonds in the silicates. On the other hand, the bands located at 488 cm^−1^ assigned to ν4 (O-Si-O) and at 690 cm^−1^ ascribed to vibrations generated by the Al-O bonds for the anhydrous slag were shifted towards lower values (at 460 and 668 cm^−1^, peaks 11 and 8, respectively). The bands appearing at 795–776, 695 and 667 cm^−1^ (peaks 5, 7 and 9) in the fly ash spectrum have all been associated with quartz and that band appearing at 554 cm^−1^ (peak 10) was assigned to mullite. Finally, in the slag spectrum, the existence of several bands at 1413, 897 and 714 cm^−1^ (peaks 2, 4 and 6) has been assigned to the presence of calcium carbonate. Of course, in both pastes, stretching and deformation modes of O-H groups of the water molecules were detected at 3500 and 1600 cm^−1^.

Between the different mixtures there were substantial changes in the region 1200–850 cm^−1^ of the spectra. The absorption bands at approximately 1200, 1100, 950, 900 and 850 cm^−1^ are associated with the Si-O-Si stretching vibrations of the SiQn units for n = 4, 3, 2, 1 and 0, respectively [46,47]. It was assumed that the band observed around 970–930 cm^−1^ was the result of the formation of C-S-H gel (Q^2^ and Q^1^ units) and the band around 1150–990 cm^−1^ was of the formation of N-A-S-H gel (Q^4^ units). The shift towards higher or lower frequencies observed in that band, indicated the predominance of one or other reaction product. For F2S8 and F4S6 mixtures, the main band was located at 982 and 999 cm^−1^ respectively, peak 3, assigned to C-S-H gel. This band was centered at higher wavenumbers than in the sample activated based solely on slag, indicating the formation of gel with a higher number of polymerized species. The IR spectra of F6S4 and F8S2 mixtures had a very intense absorption band between 1005 and 1016 cm^−1^ (peak 3) and another at 1092 cm^−1^ (peak 12). The first one corresponded to the stretching vibration in TO4 tetrahedra of N-A-S-H gel and the second indicated that the original fly ash remained without reaction. The shift of these bands to lower wavenumber with respect to the 100% fly ash paste indicated the presence of Q^2^ and Q^1^ units and a higher content of Ca in the composition of the reaction products. As the percent of fly ash increased, the spectra showed typical vibration bands of quartz (peaks 5, 7 and 9) and mullite (peak 10).

**Figure 4:**
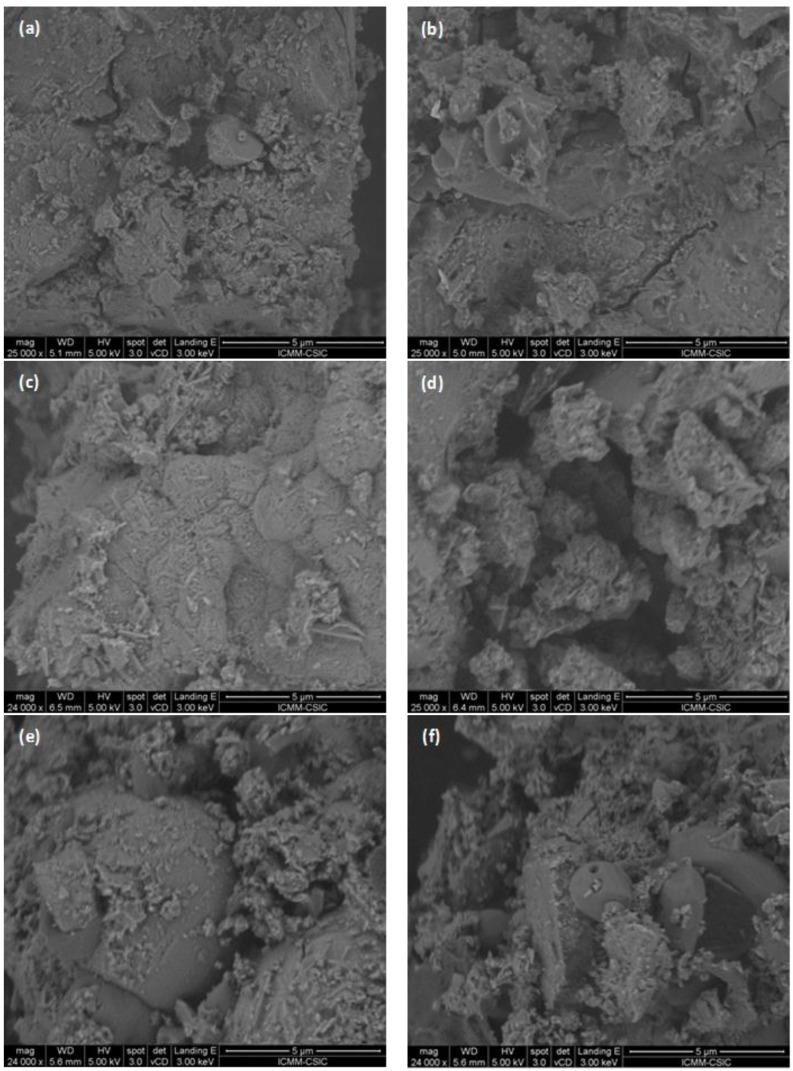
SEM images of the alkali activated mixtures: (**a**) F0S10; (**b**) F10S0; (**c**) F2S8; (**d**) F4S6; (**e**) F6S4; and (**f**) F8S2 at the age of 90 days.

The microstructure of alkali activated 100% slag and 100% fly ash pastes are shown in Figure 4a,b, respectively. Both materials had a solid matrix with almost absence of pores and very uniform structure. The superficial continuity could develop mechanical consistency. However, some fly ash particles that have not yet reacted or the presence of tracks of ash spheres were observed in F10S0 paste, interrupting the continuity of the matrix and being able to cause a decrease in mechanical strength of the material. Moreover, different microstructures were developed as a consequence of the reactive processes that happen between the slag and fly ash mixtures and the alkaline solution (Figure 4). In Figure 4c, the micrograph of the F2S8 paste shows the formation of a main reaction product with a globular morphology. The matrix was very compact and exhibited a surface coherence, while the microstructure of the F4S6 mixture presented a greater number of randomly precipitating individual particles (see Figure 4d), but the globular morphology of the reaction product was maintained. In Figure 4f, the micrographs of the F6S4 and F8S2 mixtures also show a less dense matrix than that of the F2S8 paste. In these matrixes some ash spheres partially covered with reaction product were observed. This product corresponded to a different gel that obtained for the mixtures with a higher content of slag (80% and 60%) because its morphology was totally different, like a monolayer of product and was less space-filling. Logically, in the F8S2 paste there was the greatest number of particles of unreacted fly ash.

Table 3 shows the average content of the main elements and their ratios in the reaction products in the alkali activated mixtures. The values given in this table represented an average of at least 20 individual EDX analyses. The reaction product for the four binders mainly consisted of Si, Al, Na and Ca. The differences in the content and ratio of the main elements as a function of the blend composition indicated the formation of reaction products with different compositions and structures [23,25,47], according to that observed by SEM. The main product of alkali activated F0S10 was calcium silicate hydrate with Al in its structure (C-A-S-H gel) and the average content of Al was 5.27%. In the alkali activated F10S0 paste the formation of an alkali aluminosilicate hydrate gel (N-A-S-H gel) took place; this gel presented an average content of Ca of 0.47%. It was clearly shown in Table 3 that with the increase of the fly ash content in the blend, Ca/Si and Si/Al ratios decreased while Al/Ca ratio increased. However, based only on EDX analysis results, it could not be asserted whether in these mixtures coexisted C-A-S-H and N-A-S-H phases or one hybrid C-N-A-S-H gel existed as reported previously [48,49]. For F2S8 mixture, the main reaction product had atomic ratios close to Si/Al = 2.25, Ca/Si = 0.43 and Al/Ca = 1.04, the most similar to those of the product of alkali activated 100% slag sample, type C-A-S-H gel (see Table 3). The reaction product of the alkali activated F8S2 mixture had a chemical composition clearly different with Si/Al, Ca/Si and Al/Ca ratios 1.79, 0.27 and 2.05 respectively, indicating the formation of a gel richer in Al and poorer in Ca, type N-A-S-H gel. The low Ca/Si ratio exhibited by this gel was consistent with the FTIR spectrum, which suggested the reduction of calcium concentration within the binding phase and a higher degree of polymerization. For F4S6 and F6S4 binders, their Si/Al and Ca/Si ratios were 2.05–1.98 and 0.37–0.33, respectively, a chemical composition clearly different between the alkali activated slag and fly ash types of gel products. This could indicate the formation of one hybrid C-N-A-S-H gel or the coexistence of N-A-S-H and C-A-S-H phases.

**Figure 5:**
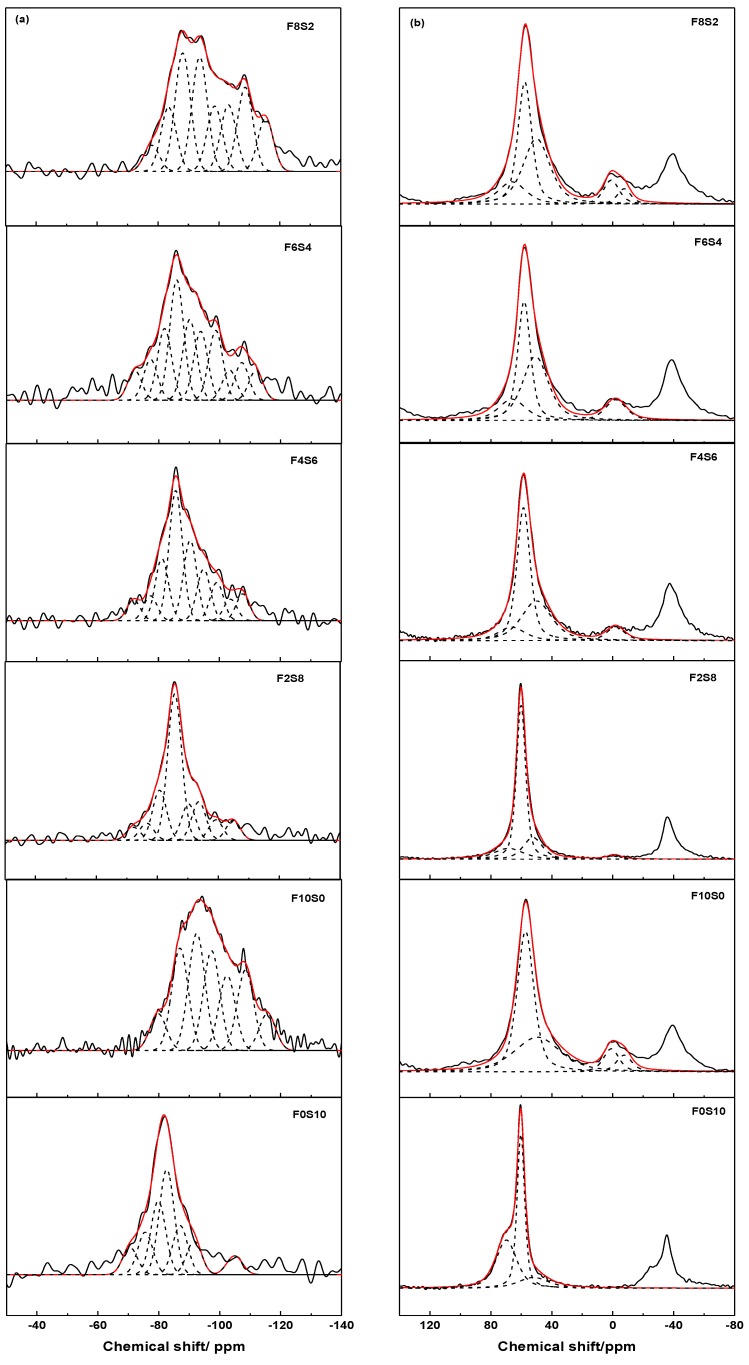
(**a**) ^29^Si; and (**b**) ^27^Al MAS-NMR spectra of the alkali-activated mixtures.

Figure 5a shows ^29^Si MAS-NMR spectra of the activated pastes, where new peaks were detected in comparison to the anhydrous GBFS and FA (see Figure 2a). The spectrum for F0S10 paste contained a wide profile with several peaks. Its deconvolution revealed the presence of unreacted Q^0^ (−71 ppm) and Q^1^ (−75 ppm) species from the raw slag. Moreover, other peaks were detected at −80, −83, −87 and −92 ppm and associated with Q^1^, Q^2^(1Al), Q^2^(0Al) and Q^3^(1Al) species, respectively. These units were attributed to the formation of an Al-substituted C-S-H type (C-A-S-H gel), as has been shown earlier by SEM [39,50]. The spectrum for F10S0 paste showed a peak at −80 ppm associated with the presence of Q^1^ (end the chain) units and other peaks that appeared at −87, −92, −97, −102, −108 ppm were attributed to Q^4^(4Al), Q^4^(3Al), Q^4^(2Al), Q^4^(1Al) and Q^4^(0Al) [51,52,53]. These five components indicated the formation of an alkaline aluminosilicate hydrate reaction product. The peak at −108 ppm was very intense and sharp, indicative that the area of the signal had a contribution of a crystalline phase of silica of the starting material. In both samples, a peak appearing around −105 ppm for the slag and −116 ppm for the fly ash was detected and assigned to different crystalline phases of silica (Q^4^(0Al) species).

In the activated mixtures, the spectra showed an important structural rearrangement and two different type of reaction products or a highly crosslinked C-N-A-S-H type gel could be detected [48,49]. The intensity and the shift of the maximum of the spectra were changed according to the percentage of the slag and fly ash in the system, see Figure 5a and Table 4. The maximum of the spectrum was −86 ppm for the mixtures with 80%, 60% and 40% of slag, while two maxima at −88 and −94 ppm were observed for the F8S2 mixture. The signals located around −77, −81, −86 and −90 ppm were associated with Q^1^, Q^2^(1Al), Q^2^(0Al) and Q^3^(1Al) units in C-A-S-H gel and the peaks detected at −94, −99, −104 and −108 ppm attributed to Q^4^(3Al), Q^4^(2Al), Q^4^(1Al) and Q^4^(0Al) units in N-A-S-H gel for F2S8 and F4S6 mixtures. In the others mixtures (with 60% and 80% of fly ash), an increase in the amount of the fly ash caused the decrease of the area of the signals (lower percentage) attributed to C-A-S-H gel and even their disappearance (Q^2^(0Al) and Q^3^(1Al) species), see Table 4. In these cases, the signal located at −90 ppm was associated with Q^4^(4Al) units in N-A-S-H gel because the profile of the spectra was more similar to that obtained in the 100% fly ash mixture, especially for F8S2 mixture. N-A-S-H gel formed was more polymerized (higher presence and percentage of Q^4^(nAl) species). In addition, as in the case of the F10S0 paste, a new signal above −111 ppm was observed in these two mixtures and associated to phases of silica (Q^4^(0Al) units). For the F2S8, F4S6 and F6S4 mixtures, the peak located at −72 ppm was associated with a mixture of Q°/Q^1^ species from the raw slag.

^27^Al MAS-NMR spectra changed substantially after the activation of the slag/fly ash mixtures, see Figure 5b. In the activated slag, three signals were observed at +69.8, +60.3 and +52.0 ppm, respectively associated with the tetrahedral aluminum (Al_T_) in the anhydrous slag, in the C-A-S-H gel and in the aluminosilicate hydrate, detected in the XRD pattern. ^27^Al MAS-NMR spectra from the activated fly ash displayed four signals centred at +57.2, +49.4, +0.9 and −7.5 ppm. The former two signals were associated to Al_T_ in the N-A-S-H gel and in the anhydrous fly ash, respectively, while the latter two signals to octrahedral aluminum in mullite [54]. The analysis of the activated mixtures confirmed that the amount of the anhydrous slag declined with an increase of the percentage of the fly ash, while the amount of the anhydrous fly ash and mullite grew, see Table 4. The width of the spectrum profile increased with increasing amount of the fly ash. For F8S2 mixture a new signal at −7.5 ppm was observed in the spectrum, associated with the AlO found in mullite. Moreover, the signal characteristic of AlT of the main reaction products (gels) was shifted downward (greater chemical shift) when the gel formed was more similar to the C-A-S-H gel and upward (smaller chemical shift) when it was more similar to the N-A-S-H gel. For F4S6 and F6S4 mixtures, this signal appeared around +58.5/+58.2, the chemical shift was intermediate indicating possible formation of a hybrid C-N-A-S-H gel.

### 3.3. Mechanical Characterization of the Alkali-Activated Binders

**Figure 6:**
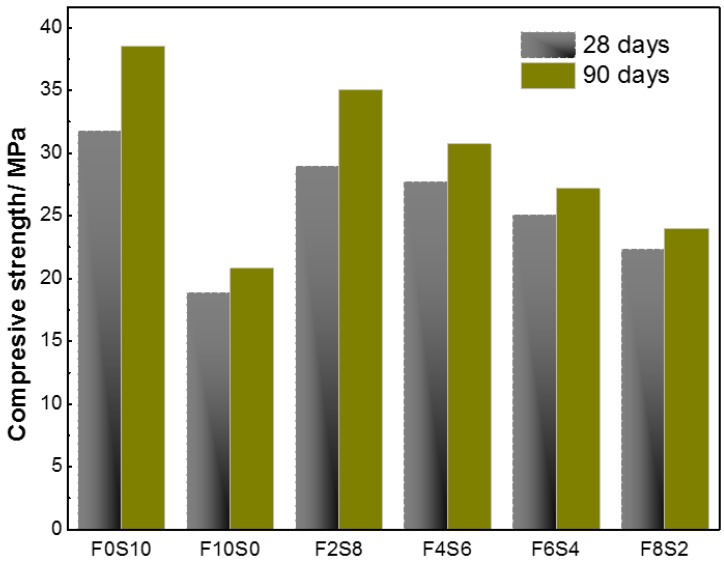
Compression strength development of alkali-activated slag/fly ash concretes over time.

The compressive strength development of alkali activated slag/fly ash concretes at the ages of 28 and 90 days is shown in Figure 6. Compressive strength of all the blends increased over the time, between 7.6% and 21.3% higher at 90 days. The gels are responsible of the mechanical development of these materials. With the curing time, a higher amount of gels was formed and higher mechanical strengths were obtained. Another feature was that the mixtures containing slag generated better performance, so their chemical characteristics and especially their content (ratio) in slag had a great influence on concrete strength at any age. A higher content of slag involved higher mechanical strengths, 31.79 and 38.55 MPa at 28 and 90 days for F0S10 concrete. To keep in mind mechanical properties of alkaline activate blends depend strongly on the characteristics of the continuous precipitate that interconnect unreacted slag and fly ash particles. F6S4 and F8S2 presented less continuity of connection between the particles (see Figure 4e,f) and therefore a considerable decrease in mechanical performance of these concretes.

**Figure 7:**
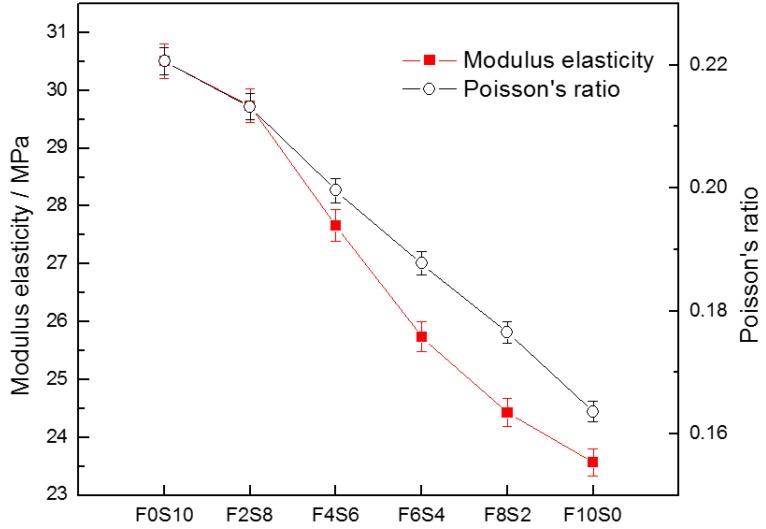
Modulus elasticity and Poisson’s ratio of the alkali-activated concretes.

Figure 7 shows the modulus of elasticity for each specimen tested. The effort was calculated as 40% of the maximum load in compression (linear behavior). The alkali activated 100% slag concrete exhibited the highest modulus of elasticity value, 30.51 GPa. The incorporation of fly ash in the slag concrete implied a decrease of the Young’s modulus, exhibiting a low ductility and toughness compared to the specific slag. F10S0 concrete had a modulus of elasticity of 23.56 GPa and deformed less in the linear range and therefore this concrete was the most rigid (less deformation with low loads).

Figure 7 also presents the values of Poisson’s ratio for the mixtures investigated. It can be observed that these values for all binders were between 0.16 and 0.22, which were quite similar to the values assigned for normal strength OPC concrete (0.11–0.21) [28]. F0S10 concrete showed the highest values of Poisson’s ratio, so these values increase with the increase of compressive strength and therefore the 100% fly ash concrete had the lowest values.

## 4. Summary and Conclusions

Based on the results presented in this work, it is reasonable to assume that the type and nature of the starting materials used directly affect to the final physical and chemical properties of geopolymers derived from waste materials. The mineralogy, microstructure, compressive strength, modulus of elasticity and Poisson’s ratio of the investigated alkali activated binders highly depended on the blend composition.

In all mixtures, two different types of reaction products were detected. For F2S8 and F4S6 mixtures, the main gel was silicate hydrate with Al in its structure (C-A-S-H gel) with a variety of environments, Q^1^, Q^2^(1Al), Q^2^(0Al) and Q^3^(1Al), rich in Ca and poor in Al and was also formed sodium aluminosilicate hydrate (N-A-S-H gel) constituted by Q^4^(3Al), Q^4^(2Al), Q^4^(1Al) and Q^4^(0Al) units. For F6S4 and F8S2 mixtures, the main gel had a higher number of polymerized species, Q^4^(4Al), Q^4^(3Al), Q^4^(2Al), Q^4^(1Al) and Q^4^(0Al), assigned to N-A-S-H gel with low content Ca and rich in Al. In addition, Q^1^, Q^2^(1Al) units were also detected, associated with C-A-S-H gel.

The slag addition reduced porosity, the matrix was very compact with better space-filling properties of C-A-S-H gel formed through the activation of slag, compared with the sodium aluminosilicate hydrate gel formed through the reaction of fly ash. The solid matrix gave way to good mechanical development of the material. The significant strength increase of the mixtures with the increased contents of slag was due to the strong load-bearing C-A-S-H type gel formed. The incorporation of slag implied that the materials exhibited a low ductility and toughness, higher deformation with high loads. Optimal characteristics of investigated alkali activated blends (high compressive strength, modulus of elasticity and Poisson’s ratio) were achieved with 80% slag and 20% fly ash mixture.

## Figures and Tables

**Table 1:** Chemical composition (% mass) of the tested fly ash (FA) and granulated blast-furnace slag (GBFS).

**Material**	**L.o.I ^a^**	**SiO_2_**	**Al_2_O_3_**	**Fe_2_O_3_**	**CaO**	**MgO**	**SO_3_**	**MnO**	**K_2_O**	**P_2_O_5_**
FA	4.93	60.6	22.9	6.8	1.0	0.6	---	---	1.5	0.4
GBFS	0.95	34.8	15.5	2.4	37.4	2.3	1.4	3.8	0.4	0.2
**Material**	**Cl**	**TiO_2_**	**V_2_O_5_**	**Rb_2_O**	**SrO**	**ZrO**	**Cr_2_O_3_**	**CuO**	**ZnO**	**---**
FA	---	1.0	---	---	0.07	0.02	0.03	0.02	0.02	---
GBFS	0.06	0.5	0.06	0.04	0.07	0.02	---	---	---	---

^a^ = Loss on Ignition.

**Table 2:** Mixture compositions.

Sample	Binder Material
F0S10	100% granulated blast-furnace slag
F10S0	100% fly ash
F2S8	20% fly ash and 80% slag
F4S6	40% fly ash and 60% slag
F6S4	60% fly ash and 40% slag
F8S2	80% fly ash and 20% slag

**Table 3:** The average content of the main elements (at %) and their ratios in the matrix of alkali-activated binders.

Blend	Si	Al	Na	Ca	Si/Al	Ca/Si	Al/Ca
F0S10	13.41 ± 0.32	5.27 ± 0.15	10.66 ± 0.13	10.80 ± 0.17	2.55 ± 0.13	0.80 ± 0.01	0.49 ± 0.02
F10S0	22.64 ± 1.14	9.50 ± 0.34	10.40 ± 0.08	0.47 ± 0.13	2.39 ± 0.20	0.02 ± 0.00	20.94 ± 6.64
F2S8	15.58 ± 1.12	6.93 ± 1.59	9.88 ± 0.12	6.64 ± 0.29	2.25 ± 0.59	0.43 ± 0.09	1.04 ± 0.68
F4S6	15.46 ± 2.87	7.52 ± 1.01	9.11 ± 2.71	5.81 ± 2.09	2.05 ± 0.66	0.37 ± 0.21	1.29 ± 0.31
F6S4	16.81 ± 0.08	8.50 ± 0.20	11.40 ± 1.11	5.49 ± 1.89	1.98 ± 0.05	0.33 ± 0.11	1.55 ± 0.61
F8S2	16.76 ± 0.90	9.37 ± 1.29	10.73 ± 0.32	4.57 ± 1.20	1.79 ± 0.15	0.27 ± 0.09	2.05 ± 0.85

**Table 4:** ^29^Si MAS NMR data of alkali-activated mixtures.

**Mixtures**		**Raw Slag**		**End of Chain**	**C-A-S-H Gel**				**N-A-S-H Gel**					**Phases of Silica**
		**Q^0^**	**Q^1^**	**Q^1^**	**Q^1^**	**Q^2^(1Al)**	**Q^2^(0Al)**	**Q^3^(1Al)**	**Q^4^(4Al)**	**Q^4^(3Al)**	**Q^4^(2Al)**	**Q^4^(1Al)**	**Q^4^(0Al)**	**Q^4^(0Al)**
F0S10	Maximum of the spectrum (ppm)	−70.6	−75.5	---	−79.8	−82.6	−87.0	−91.6	---					−104.9
	Width	5.79	5.79		5.79	5.79	5.79	5.79						5.79
	Intermediate (%)	7.70	12.11		20.96	30.04	14.01	9.55						5.63
F10S0	Maximum of the spectrum. (ppm)	---		−79.8	---				−86.9	−92.4	−97.3	−102.4	−108.4	−115.6
	Width			6.35					6.35	6.35	6.35	6.35	6.35	6.35
	Intermediate. (%)			6.70					18.66	21.36	18.20	13.72	14.67	6.69
F2S8	Maximum of the spectrum. (ppm)	−72.1		---	−76.5	−80.7	−85.7	−90.2	---	−93.6	−99.2	−104.9	---	---
	Width	5.41			5.41	5.41	5.41	5.41	---	5.41	5.41	5.41	---	---
	Intermediate. (%)	3.80			5.00	14.63	42.81	10.62	---	11.30	5.97	5.87	---	---
F4S6	Maximum of the spectrum. (ppm)	−71.8		---	−77.4	−81.1	−85.7	−90.4	---	−94.8	−99.1	−103.6	−107.7	---
	Width	5.15			5.15	5.15	5.15	5.15	---	5.15	5.15	5.15	5.15	---
	Intermediate. (%)	4.59			5.45	13.59	28.49	17.62	---	11.32	8.37	4.70	5.86	---
F6S4	Maximum of the spectrum. (ppm)	−72.1		---	−77.2	−81.9	−85.9	---	−90.3	−94.1	−98.9	−103.9	−107.4	−111.5
	Width	5.22			5.22	5.22	5.22	---	5.22	5.22	5.22	5.22	5.22	5.22
	Intermediate. (%)	5.13			7.07	12.37	20.77	---	13.92	11.88	12.05	5.22	6.49	5.11
F8S2	Maximum of the spectrum. (ppm)	---		---	−77.9	−83.5	---	---	−88.1	−93.7	−98.6	−103.1	−108.6	−115.3
	Width				5.98	5.98	---	---	5.98	5.98	5.98	5.98	5.98	5.98
	Intermediate. (%)				4.43	10.81	---	---	19.97	19.33	11.03	11.25	14.20	8.98
